# Wnt-activating human skin organoid model of atopic dermatitis induced by *Staphylococcus aureus* and its protective effects by *Cutibacterium acnes*

**DOI:** 10.1016/j.isci.2022.105150

**Published:** 2022-09-16

**Authors:** Song-yi Jung, Hyun Ju You, Min-Ji Kim, GwangPyo Ko, Seunghee Lee, Kyung-Sun Kang

**Affiliations:** 1 Adult Stem Cell Research Center and Research Institute for Veterinary Science, College of Veterinary Medicine, Seoul National University, Seoul 08826, Republic of Korea; 2 Department of Environmental Health Sciences, Graduate School of Public Health, Seoul National University, Seoul 08826, Republic of Korea; 3 Bio-MAX/N-Bio, Seoul National University, Seoul 08826, Republic of Korea; 4 KoBioLabs, Inc., Seoul 08826, Republic of Korea; 5 Stem Cell and Regenerative Bioengineering Institute, Global R&D Center, Kangstem Biotech Co. Ltd., Seoul 08590, Republic of Korea

**Keywords:** Pathophysiology, Microbiology, Stem cells research

## Abstract

A recently developed human PSC-derived skin organoid model has opened up new avenues for studying skin development, diseases, and regeneration. The current model has limitations since the generated organoids are enclosed, circular aggregates with an inside-out morphology with unintended off-target development of cartilage. Here, we first demonstrated that Wnt signaling activation resulted in larger organoids without off-target cartilage. We optimized further using an air-liquid interface (ALI) culture method to recapitulate structural features representative of human skin tissue. Finally, we used the ALI-skin organoid platform to model atopic dermatitis by *Staphylococcus aureus* (SA) colonization and infection. SA infection led to a disrupted skin barrier and increased production of epidermal- and dermal-derived inflammatory cytokines. Additionally, we found that pre-treatment with *Cutibacterium acnes* had a protective effect on SA-infected organoids. Thus, this ALI-skin organoid platform may be a useful tool for modeling human skin diseases and evaluating the efficacy of novel therapeutics.

## Highlights

• Wnt signaling activation results in larger organoids without off-target cartilage
• Air-liquid interface culture is used to recapitulate human skin tissue structure
• *S. aureus* infection damaged the skin barrier and elevated inflammatory cytokines
• Pre-treated *Cutibacterium acnes* had a protective effect on *Staphylococcus aureus*-infected organoids

## Graphical abstract

**Figure:**
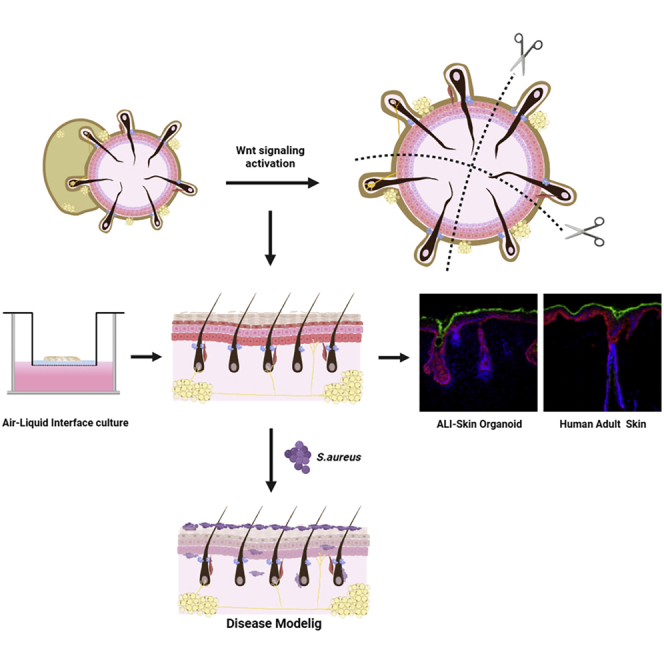

## Introduction

A hair-bearing human pluripotent stem cell (hPSC)-derived skin organoid model has been recently developed to recapitulate the complexity and function of human skin layers, nerve cells, melanocytes, and appendages, such as sebaceous glands and hair follicles, associated with normal human skin (Lee et al., 2020). It provides a new platform to investigate skin development, diseases, and regeneration. Despite the potential of a skin organoid model system, there are several challenges. The skin organoids are enclosed, circular aggregates that exhibit an inside-out morphology. The dermal and hypodermal layers are in the outer layer of the organoid, while the epidermal layer is located in the internal core of the organoid. Another morphological limitation of the skin organoid model is the unintended, off-target differentiation of hyaline cartilage in the tail region (Lee et al., 2020; Ramovs et al., 2022). While the organoid model system may be useful for many applications, it may not be optimal for the investigation of skin development and modeling of diseases of stratified organs, such as those of the skin. Moreover, variations in the properties of organoids generated by the current protocol are a major challenge for their application in drug discovery and skin disease modeling. Therefore, the current skin organoid culture system needs to be optimized further.

Microbiome analyses have revealed that the human skin is colonized by a diverse population of bacterial communities (Byrd et al., 2018). Numerous endogenous and exogenous factors can affect the symbiotic host-microorganism relationships, leading to skin infections and disorders such as atopic dermatitis (AD) (Byrd et al., 2018; Flowers and Grice, 2020; Kim and Kim, 2019; Paller et al., 2019; Schommer and Gallo, 2013). Previous studies have shown that *S. aureus*, a common cause of skin infections, is frequently found on the skin of patients with AD but not on healthy individuals (Flowers and Grice, 2020). However, it is unclear whether *S. aureus* is a direct cause of AD or a result of a disrupted epithelial barrier. Recent studies have suggested that *S. aureus* colonization and microbiome dysbiosis might contribute to the development and progression of the disease by disrupting the skin barrier and immune response (Fyhrquist et al., 2019; Kong et al., 2012; Meylan et al., 2017; Nakamura et al., 2020; Williams and Gallo, 2017). While substantial advancements have been made, the mechanisms of AD development and progression via *S. aureus* colonization and infection are poorly understood. This is particularly the case because most studies on the relationship between the skin and *S. aureus* colonization were done on small animal models, *in vitro* keratinocytes in 2D cultures, or 3D skin models, such as human skin equivalents, which rely on co-culturing primary epidermal and dermal cells with a few other skin cell types (Emmert et al., 2020; Holland et al., 2008; Rademacher et al., 2018). These model systems often fail to accurately depict the interactions between human skin, *S. aureus*, and commensal microbiota found in healthy individuals, either as a result of species-specific differences or the inability to recapitulate the complexity and organization of skin physiology.

In this study, we first modified the skin organoid protocol by Wnt activation, resulting in organoids of increased size without cartilage development. Additionally, we adapted an ALI culture method to generate skin organoids more closely resembling human skin physiology (Pruniéras et al., 1983). We show that the ALI-skin organoids have a stratified squamous epithelium, which is similar to the structure of human adult skin. Using the ALI-skin organoids, we modeled AD with *S. aureus* skin colonization and infection. We found that *S. aureus* could penetrate the epidermis and infect the dermal layer. *S. aureus*-infected ALI-skin organoids had a disrupted epidermal skin barrier and showed increased production of epidermal- and dermal-derived inflammatory cytokines, recapitulating human AD pathologies. For the first time, we demonstrate direct causality between AD and *S. aureus* colonization and infection in 3D hPSC-derived skin organoids. Also, we discovered that *C*. *acnes*, a commensal skin bacterium found on healthy human skin, protected ALI-skin organoids from *S. aureus*-mediated impairment of the skin barrier.

## Results

### Generation of hiPSC-derived skin organoid with Wnt signaling activation

First, skin organoids were successfully generated from human-induced pluripotent stem cells (hiPSCs) using the recently published hair-bearing skin organoid culture method of Lee et al. (2020) (Figure S1A). The generated skin organoids were spherical with large hyaline cartilage in the tail region of the organoids (Figures S1B, S1C, and S1D). Organoid-to-organoid variations were observed whereby each batch of organoids contained either multiple pigmented or albino hair follicles (Figure S1C). At day 120, we observed that PDGFRα^+^ dermal and oil red O staining^+^ hypodermal layers were present in the outer layer of the organoids, and the KRT17^+^ basal and peridermal layers were located in the internal core of the organoids, exhibiting an inside-out morphology at day 91 (Figure S1E). While these skin organoids are useful for many applications, they may not be applicable for the study of skin development and modeling the diseases of stratified skin. Therefore, the current skin organoid culture system needs to be optimized further.

**Figure 1:**
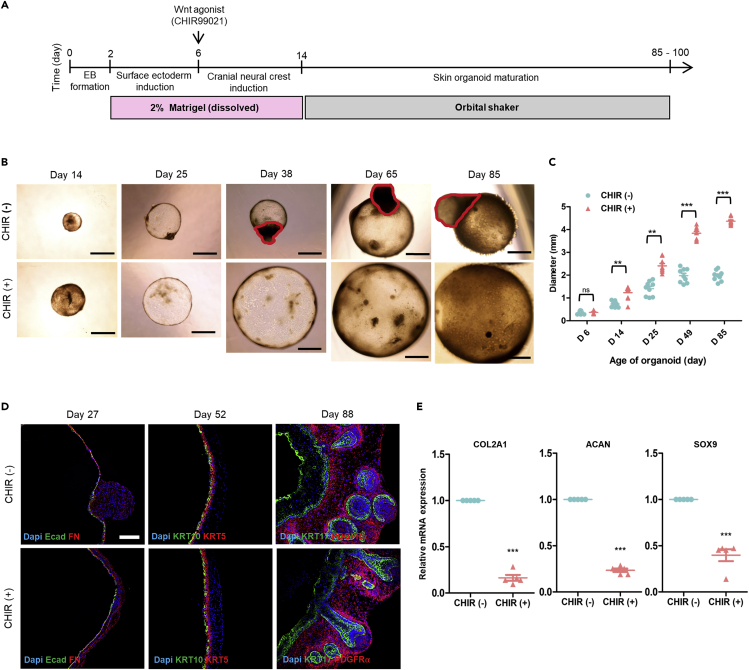
Generation of human-induced pluripotent stem cell (hiPSC)-derived skin organoids with activation of the Wnt signaling pathway (A) Schematic overview and timeline of the culture protocol for generating skin organoids from hiPSCs. Day 0 refers to when hiPSC colonies are detached to form embryoid bodies (EB). (B) Bright-field images of the same skin organoids captured at days 14, 25, 38, 65, and 85 without (CHIR-) or with CHIR (CHIR+) treatment. Scale bar, 1 mm. The red circles represent hyaline cartilages on CHIR (−). (C) Quantification of the diameter of skin organoids based on the bright-field images of whole skin organoids (n = 9, CHIR (+) versus CHIR (−), independent replicates = 3). (D) Immunostaining for epithelial (Ecadherin; Ecad) and mesenchymal (Fibronectin; FN) markers at day 27, epithelial differentiation markers (KRT 5 and KRT 10) at day 52, and epithelial (KRT17) and dermal layer (PDGFR) markers at day 88 in CHIR (−) and CHIR (+) organoids. Scale bar, 50 μm. (E) Real-time qPCR analysis of hyaline cartilage markers (COL2A1, ACAN, and Sox9) in skin organoids with or without CHIR treatment at days 60–65 (CHIR (+) versus CHIR (−). n = 5). Statistical analysis was performed using two-way ANOVA with Bonferroni post-hoc test ns: not significant, ∗∗p < 0.01, ∗∗∗p < 0.001. The results are presented as the means ± SEM.

Previous studies have demonstrated that Wnt signaling activation by the GSK3 beta inhibitor (CHIR99021) enhances organoid growth (Qian et al., 2016) and inhibits migration of cranial neural crest cells (CNCC), preventing cartilage formation (Gonzalez Malagon et al., 2018). In this study, we investigated whether Wnt signaling activation by supplementing the GSK3 beta inhibitor (CHIR99021) promotes skin organoid development and inhibits CNCC migration. First, organoids were grown similarly to the protocol described by Lee et al. In a recent study (Lee et al., 2020), hiPSCs were dissociated into single cells to form embryoid bodies (EBs) in low-attachment U-bottom 96-well dishes. To promote surface ectoderm differentiation, these EBs were treated with SMAD inhibition factor (SB431542), FGF2, and BMP4. On day 6, EBs were treated with the BMP4 inhibitor (LDN), and FGF2 for CNCC induction, as well as CHIR99021 (3 μM CHIR) to activate canonical Wnt signaling (Figure 1A). We tested different doses of CHIR to culture the organoids and found that the highest dose of CHIR (above 3 μM) did not sustain organoid survival (data not shown). Wnt signaling pathway activation by CHIR treatment at day 6 of differentiation resulted in significantly larger organoids at day 14, 25, 49, and day 85 than those without CHIR treatment (Figures 1B and 1C). Some samples in the CHIR treatment group grew as cysts with diameters of up to 7 mm after 85 days of culture (Figure 1B). Furthermore, we observed that hyaline cartilage development on skin organoids was inhibited by CHIR treatment (Figure 1B). Immunostaining analysis of skin organoid cryosections revealed the presence of Ecadherin^+^ epidermal and Fibronectin (FN)^+^ mesenchymal layers as early as day 27 of differentiation irrespective of CHIR treatment (Figure 1D). In the absence of CHIR treatment, spherical FN^+^ mesenchymal cells migrated and aggregated on the organoids. CHIR-treated organoids at day 27 had an E-cadherin^+^ epithelial layer surrounded evenly by FN. On day 52, the basal epithelial marker (KRT5) and the mature spinous epithelial marker (KRT10) were observed in a similar pattern in both groups of organoids (Figure 1D). On day 88, we observed KRT17^+^ hair follicle structures and PDGFRα^+^ dermal layers in both groups of skin organoids. The KRT17^+^ epithelial layer of CHIR-treated organoids was thinner than that of organoids cultured without CHIR treatment (Figure S1F), probably due to size differences (Figures 1B and 1C). Moreover, we confirmed by real-time qPCR analysis that hyaline cartilage development was inhibited in CHIR-treated skin organoids on day 60–65. We found significantly lower expression of chondrogenic-related genes (Sekiya et al., 2000), including COL2A1, ACAN, and SOX9, in CHIR-treated organoids than in CHIR-untreated organoids (Figure 1E). Therefore, we optimized the skin organoid protocol by activating the Wnt signaling pathway, resulting in organoids of increased size with no off-target cartilage differentiation.

### Air-liquid interface (ALI)-skin organoids

**Figure 2:**
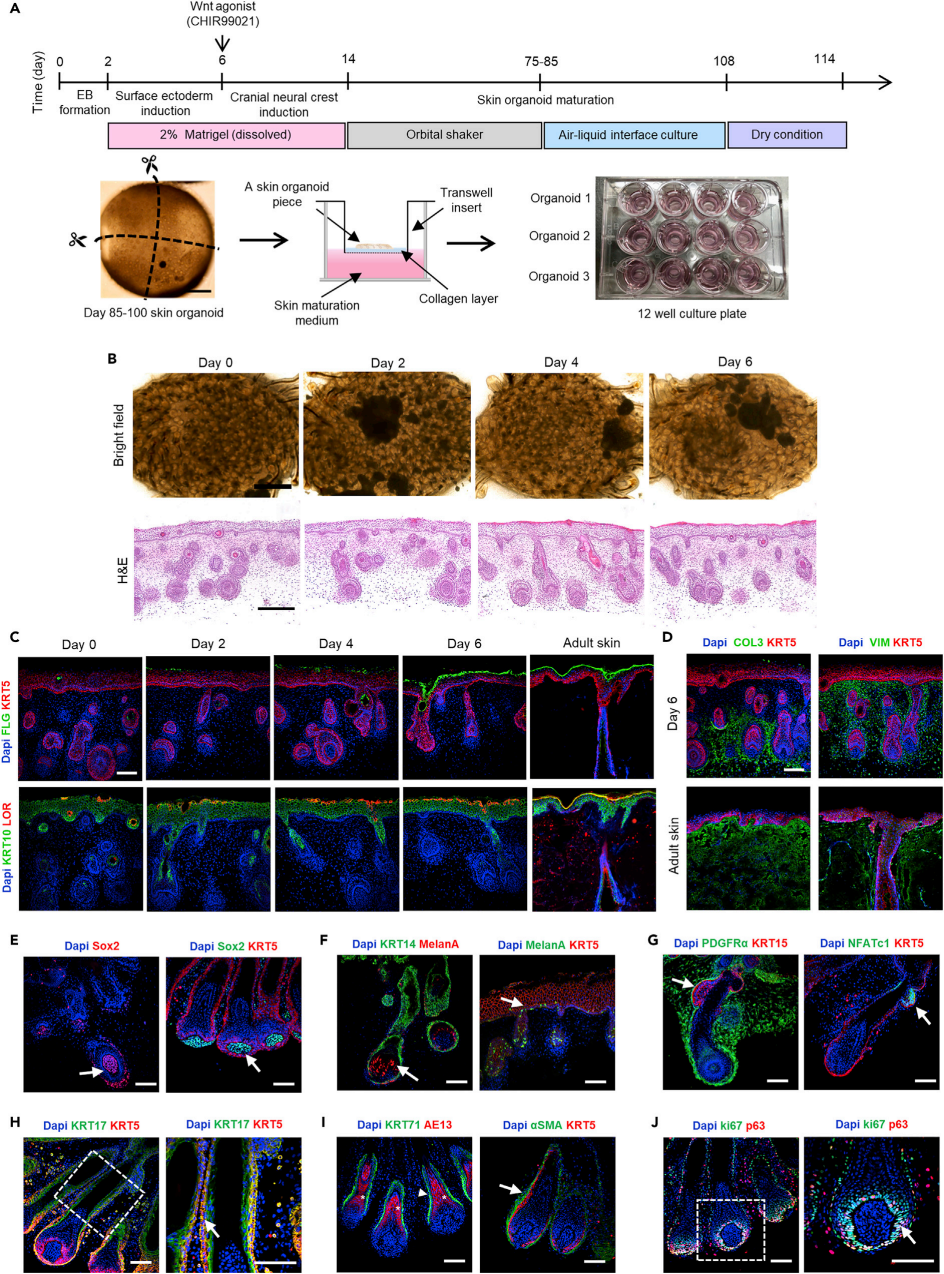
Generation of air-liquid interface (ALI)-skin organoids (A) Schematic overview and timeline of the culture protocol for generating ALI-skin organoids from hiPSCs. Day 0 refers to when hiPSC colonies were detached to form EBs and an overview of the ALI-skin organoid culture model. (B) Bright-field images of ALI-skin organoids after 0, 2, 4, and 6 days of culture in dry conditions. Scale bars, 50 μm, and H&E staining of ALI-skin organoids after 0, 2, 4, and 6 days of culture in dry conditions. Scale bars, 100 μm. (C) Immunostaining for epidermal barrier markers in ALI-skin organoids after 0, 2, 4, and 6 days of culture in dry conditions and in human adult skin. Cornified (Filaggrin; green) and basal (KRT5; red) epidermal layer markers are shown on the top, and spinous (KRT10; green) and granular (Loricrin; red) epidermal layer protein markers are characterized on the bottom panel. Scale bars, 50 μm. (D) Immunostaining for dermal layer markers (Vimentin and Collagen 3; green) and basal epithelial layer markers (KRT5; red) in ALI-skin organoids after 6 days in dry conditions (top panel) and in human adult skin (bottom panel). Scale bars, 50 μm. (E–J) Hair follicle characterization in ALI-skin organoids. Shown are immunostaining images for Sox2^+^ dermal papilla (arrows; left and right) (E), melanocytes (melanA) found in elongated hair follicles (arrow; left) and epidermal layer (arrow; right) (F), KRT15^+^KRT5^+^ bulge stem cells (arrow; left) and NFATc1^+^KRT5^+^ bulge stem cells (arrow; right) (G), KRT17^+^KRT5^+^ outer sheaths of hair shafts; dashed box, magnified region (arrow; right) (H), KRT71^+^ inner sheaths of hair shafts (arrowhead; left), AE13^+^ hair shaft cortex (asterisk; left), and ⍺SMA^+^KRT5^+^ dermal sheath (arrow; right) (I), and ki67^+^p63^+^ hair matrix cells, dashed box, magnified region (arrow; right) (J). Scale bars, 50 μm.

To establish a skin organoid model resembling more closely that of human skin, we utilized an ALI culture method (Pruniéras et al., 1983) (Figure 2A). Large skin organoid cysts without off-target differentiation of cartilage are optimal for ALI culture. The ALI culture method employs a Transwell permeable support where the basal surface of the cells is in contact with the liquid culture medium, whereas the apical surface is exposed to the air to increase oxygenation (Figure 2A). This ALI culture method was adapted from a recent PSC-derived cerebral organoid culture study to generate organoids with improved viability, maturation, and function (Giandomenico et al., 2019). For the ALI-skin organoid culture, ALI culture was started from around day 85 to day 100. Day 85 was chosen because the differentiation of the Loricrin^+^ and Filaggrin^+^ mature epithelial layers starts to occur around 85 (Figure S2A), and the organoids were thick enough to cut into pieces and easy to handle for ALI culture. In this study, each organoid was dissected into four evenly sized portions that were cultured individually on top of a collagen-coated Transwell permeable support in a 12-well plate format, so that each skin organoid could be tested in four different experimental conditions (Figure 2A). The dermal layer was placed face-down on top of a cellular collagen-coated insert so that the epidermal layer was exposed to the air. This culture format not only reduces variation among individual organoids but also improves maturation of the epidermal layers and hair follicle growth. The optimal time for epithelial skin maturation was first investigated after 3 weeks of ALI culture in a 100% humidified incubator. Subsequently, the skin organoids were cultured by incubating at 37°C with 5% CO_2_ without humidification (dry condition) for 0, 2, 4, and 6 days to mature the keratinocytes of the skin organoids. Bright-field images of each ALI-skin organoid were taken after 0, 2, 4, and 6 days of dry culture conditions (Figure 2B). Histologically, the ALI-skin organoids developed into continuous epithelial structures, resulting in the efficient formation of mature stratified epithelial layers that had a normal basket weave pattern on their surfaces, characteristic of cornified stratum (Williams and Elias, 1993). We found significantly increased cornified stratum formation on day 4 and day 6 of dry culture conditions (Figure 2B; H&E staining and Figure S2B). To examine the stratified epithelial structures in more detail, immunostaining analysis was performed, which revealed that on day 0 of dry culture condition, basal (KRT5) and spinous (KRT10) epidermal layers were fully differentiated in the ALI-skin organoids (Figure 2C). Expression of loricrin and filaggrin, considered as late markers of epidermal differentiation, was observed in the ALI-skin organoids starting from day 2 of dry condition culture. On day 6 of the dry culture condition, we observed loricrin and filaggrin positive fully differentiated granular and cornified layers of the epidermis in the ALI-skin organoid, respectively (Figure 2C). We determined the expression levels of loricrin and filaggrin by calculating the mean fluorescence intensities and found that the expression levels of loricrin and filaggrin were significantly elevated on day 6 under dry culture conditions (Figures S2C and S2C). In addition, the expression of loricrin and filaggrin on day 6 of dry culture conditions was compared to that of adult human skin. We discovered that the expressions of loricrin, filaggrin, and KRT10 in ALI-skin organoids after six days of culture under dry conditions were comparable to those in adult human skin (Figure 2C). As observed in adult human skin, type 3 collagen (COL3A) and vimentin (VIM) were localized to the dermal extracellular matrix protein in the dermal layer (Figure 2D). Six days of dry culture of ALI-skin organoids revealed the presence of LipidTox-positive lipids in the hypodermal layer and sebaceous glands (Figure S2E). Following this, we examined hair follicles in ALI-skin organoids as described by Lee et al. Expression of Sox2^+^ dermal papilla (Figure 2E) and melanA^+^ melanocytes was observed in elongated hair follicles as well as the epidermis of ALI-skin organoids (Figure 2F). We found that KRT15^+^ KRT5^+^ and NFATc1^+^ and KRT5^+^ hair follicle bulge stem cells were also present (Figure 2G). Robust and defined expressions of KRT17^+^ KRT5^+^ outer sheaths of hair shafts were expressed as well in the ALI-skin organoids (Figure 2H). Additionally, KRT71^+^ inner sheaths of hair shafts and AE13^+^ cuticles were observed (Figure 2I). Alpha SMAα^+^ KRT5^+^ dermal sheaths were observed on the hair follicles of ALI-skin organoids (Figure 2I). In addition, a ki67^+^ p63^+^ hair follicle matrix was found (Figure 2J). Consequently, the culture of ALI-skin organoids in dry conditions for six days was sufficient to differentiate mature keratinocytes, resembling the skin of an adult human and develop hair follicles.

### Modeling of AD by *S. aureus* colonization and infection of ALI-skin organoids

**Figure 3:**
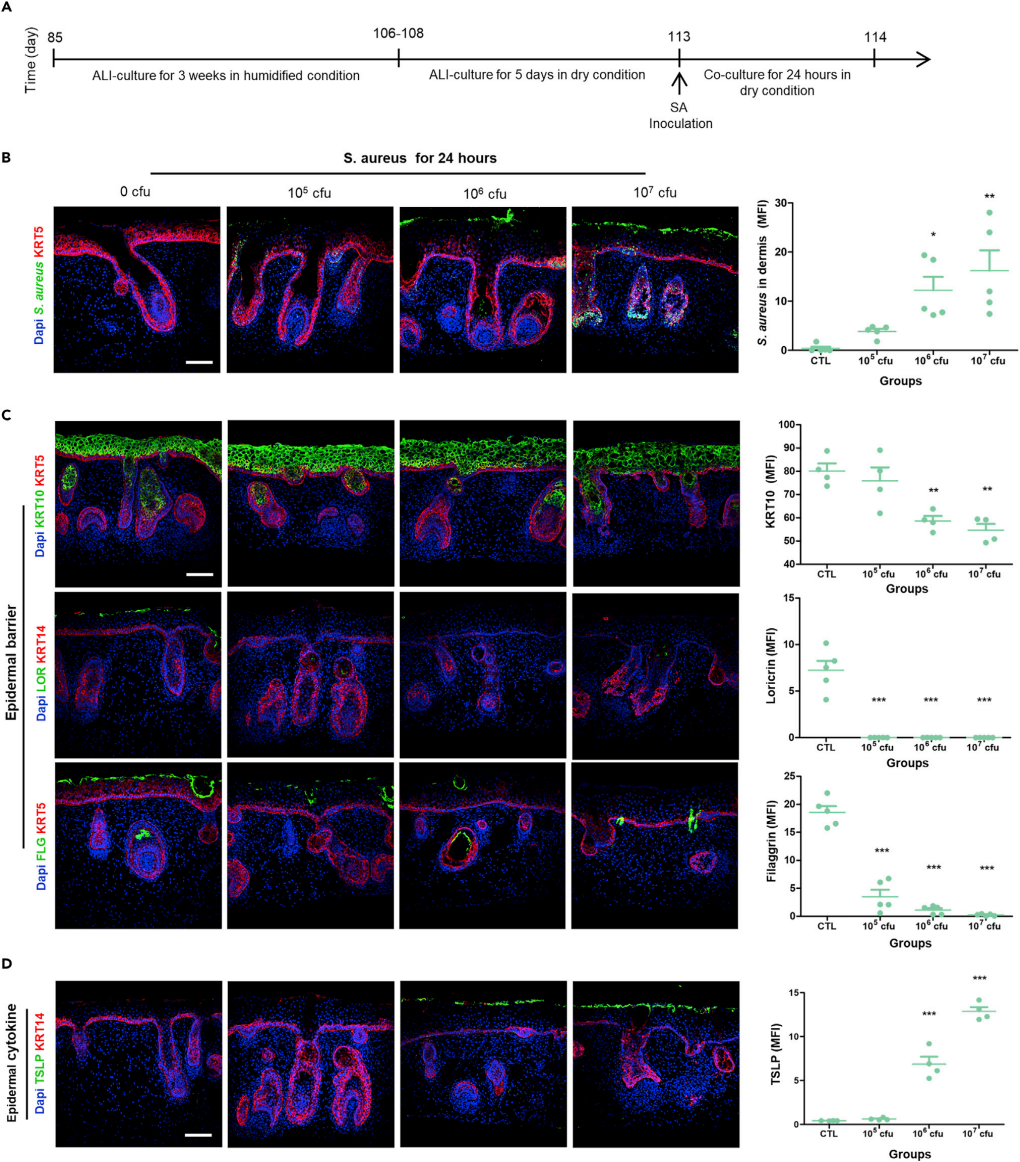
Modeling of atopic dermatitis (AD) by *S. aureus* colonization and infection of ALI-skin organoids (A) Schematic outline of a *S. aureus* and ALI-skin organoid co-culture method. (B) *S. aureus* immunostaining to visualize bacteria and the KRT5^+^ basal epithelial layer maker (red) in 0 CFU (control; PBS only), 10^5^ CFU, 10^6^ CFU, or 10^7^ CFU *S. aureus*-infected ALI-skin organoids (left) and *S. aureus* infection in the dermal layer as mean fluorescence intensity (MFI) was analyzed in each group (right; independent replicates = 5). Scale bars, 50 μm. (C) Epidermal barrier markers were analyzed in ALI-skin organoids infected with 0 CFU, 10^5^ CFU, 10^6^ CFU, or 10^7^ CFU SA. Immunostaining images for KRT5^+^ basal and KRT10^+^ spinous epithelial layer markers (left) and quantification of KRT10 expression level (MFI; right) on the top panel, immunostaining images for LOR^+^ granular layer marker (left) and its quantification analysis (MFI; right) on the middle panel, and immunostaining images for FLG^+^ cornified epidermal layer marker (left) and its quantification analysis (MFI; right) on the bottom panel are shown. One-way ANOVA with Bonferroni post-hoc test; ∗∗∗p < 0.001, compared to 0 CFU; independent replicates = 5. Scale bars, 50 μm. (D) Immunostaining images for TSLP in green and KRT14 in red in ALI-skin organoids infected with 0 CFU, 10^5^ CFU, 10^6^ CFU, or 10^7^ CFU *S aureus* (left) and quantification of TSLP expression on the epidermal layer (MFI; right; independent replicates = 4). Scale bars, 50 μm. Statistical analysis was performed using two-way ANOVA with Bonferroni post-hoc test ∗p < 0.05, ∗∗p < 0.01, ∗∗∗p < 0.001. The results are presented as the means ± SEM.

Recent microbiome studies have revealed that 95% of patients with AD are colonized with *S. aureus* (Fyhrquist et al., 2019). We first focused on whether AD pathological characteristics could be recapitulated in ALI-skin organoids infected with *S. aureus* and whether these infected ALI-skin organoids could be used as a model of AD. First, a series of experiments were performed to assess the transient effect of *S. aureus* on human skin by inoculating bacteria on the surface of the ALI-skin organoids. The ALI-skin organoid culture was started around day 85 (Figure 3A) in a humidified incubator. After 3 weeks, the ALI-skin organoids were matured in dry conditions for 6 days with skin maturation media without antibiotics. Bacterial strains may grow at different rates in ALI-skin organoid culture conditions and this could influence their interactions with the skin. Thus, different doses (0, 10^5^, 10^6^, and 10^7^ CFU) of *S. aureus* were inoculated on the surface of the ALI-skin organoids and co-cultured for 24 h (Figure 3A). Immunostaining against *S. aureus* was performed on ALI-skin organoids to determine whether *S. aureus* actively penetrated the skin barrier (Figure 3B). Significant amounts of *S. aureus* were detected in the dermal layer of 10^6^ and 10^7^ CFU *S*. *aureus*-infected ALI-skin organoids, according to quantitative analysis (Figure 3B). *S. aureus* penetrated the epithelial surface layer and dermal layer of the skin organoid, indicating that it actively infiltrates human skin organoids. Similarly, structural damage was observed in the epidermal and dermal layers of *S. aureus*-infected ALI-skin organoids (Figure S3A; H&E staining). We next focused our attention on whether epidermal barrier disruption, a hallmark of AD pathology (Hanifin, 2018; Weidinger et al., 2018), is recapitulated in the *S. aureus*-infected ALI-skin organoids. Immunostaining analysis revealed that *S. aureus*-infected ALI-skin organoids had significantly reduced expression of KRT10 (spinous) epidermal layer markers in 10^6^ and 10^7^ CFU *S aureus*-infected ALI-skin organoids (Figure 3C). Expression of Loricrin (LOR), a granular epidermal layer marker, was diminished after 10^5^ CFU *S aureus* infection (Figure 3C). Moreover, expression of filaggrin, a cornified layer marker, was reduced significantly in a dose-dependent manner when compared to the 0 CFU *S. aureus*-infected ALI-skin organoids (Figure 3C). Furthermore, infection of ALI-skin organoids with *S. aureus* for 24 h decreased the expression of an important tight junction protein marker, Claudin 4 (Figure S3B). These results indicate that *S. aureus* infection disrupted the epithelial barrier of the ALI-skin organoids. This is similar to the AD phenotype that has already been described (De Benedetto et al., 2011; McAleer and Irvine, 2013). The expression of epidermal stem cell markers, KRT5^+^ KRT15^+^ and KRT5^+^ p63^+^, also decreased in *S. aureus*-infected ALI-skin organoids (Figure S3C). Importantly, thymic stromal lymphopoietin (TSLP) is one of the major epithelial cell-derived inflammatory cytokines known to play a role in AD (Wilson et al., 2013). Immunostaining analysis revealed that TSLP induction was observed on the surface of the epithelial cell layers of 10^6^ and 10^7^ CFU *S. aureus*-infected ALI-skin organoids (Figure 3D). We also found a significant increased level of TSLP in *S. aureus-*infected ALI-skin organoid culture supernatants (Figure S3D). Together, the occurrence of a disrupted skin barrier, decreased epidermal stem cells, and induction of epithelial-derived cytokines suggests that the *S. aureus*-infected ALI-skin organoid model of AD recapitulates human disease. Furthermore, the proliferation (Ki67) and apoptotic cell death (TUNEL) markers were assessed by immunostaining the *S. aureus-*infected ALI-skin organoids cryosections (Figure S3E). We found that *S. aureus* at 10^7^ CFU drastically increased the number of TUNEL assay positive cells in the ALI-skin organoids and thereby caused substantial cell death (Figure S3E). The number of Ki67 + cells significantly decreased only in 10^7^ CFU of *S. aureus*-infected ALI-skin organoids. However, proliferation and apoptosis rates did not differ between the control (0 CFU), 10^5^ CFU, or 10^6^ CFU *S. aureus*-infected ALI-skin organoids. Thus, only the 10^7^ CFU *S. aureus*-infected ALI-skin organoid group had increased cell death and reduced cell proliferation compared with the lower *S. aureus* CFU groups. Therefore, the above observed effects were not the results of increased cell death and reduced cell proliferation except for the 10^7^ CFU *S. aureus*-infected ALI-skin organoid group.

### Transcriptome analysis of *S. aureus*-infected ALI-skin organoids

**Figure 4:**
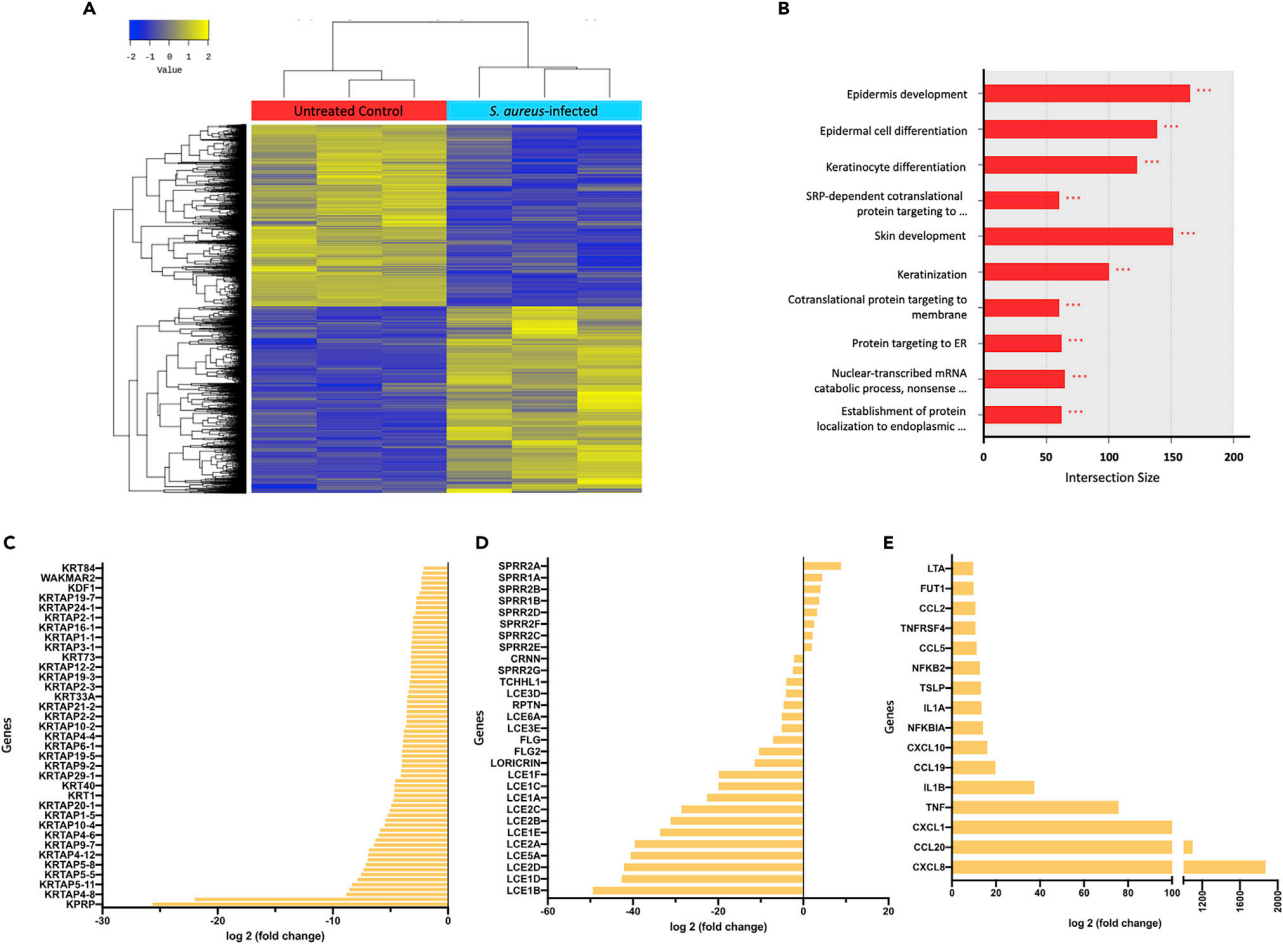
Transcriptome analysis of *S. aureus*-infected ALI-skin organoids (A) The differentially expressed genes (DEGs) are visualized. A heatmap of the one-way hierarchical clustering using *Z* score for normalized value (log2 based) (4,255 genes satisfying fc2 and raw. P) is shown. Genes in untreated control (in red) and genes in *S. aureus*-infected (blue) ALI-skin organoids are shown. (B) The DEGs were classified based on the top 10 terms of GO functional analysis of biological processes (∗p < 0.05, ∗∗p < 0.01, ∗∗∗p < 0.001). (C and D) Downregulated genes involved in keratinocyte differentiation genes (C) and in epidermal barrier functional genes (D) by *S. aureus* infection are shown. (E) Upregulated genes for the skin organoid derived inflammatory markers by *S. aureus* infection are shown.

We next investigated transcriptional changes associated with *S. aureus* infection by performing RNA sequencing. After 18 h of co-culture with *S. aureus*, the transcriptome analysis revealed significant cellular responses in ALI-skin organoids. Hierarchical clustering and MDS analysis depicted distinct differences in the expression of a large number of genes between the biological replicates of *S. aureus*-infected ALI-skin organoids and uninfected controls (Figure S4A). The differentially expressed genes (DEGs) were visualized using a volcano plot and a heatmap depicting altered genes (Figure 4A and S4B). Of the 4,255 DEGs, 2,162 genes were upregulated and 2,093 genes were downregulated by *S. aureus* inoculation (with adjusted p value <0.05). To better understand the changes in transcriptomes after *S. aureus* infection, the DEGs were classified based on the GO terms in biological processes (Figure 4B). The DEGs were represented by various metabolic processes, in particular, the regulatory functions of epidermis development, epidermal cell differentiation, keratinocyte differentiation, keratinization, and cornified envelope differentiation (Figure 4B). We also found that genes related to skin barrier function and keratinocyte differentiation, such as FLG (filaggrin), LCE (late cornified envelope), Loricrin, SPRR (small proline rich protein), RPTN (repetin), CRNN (cornulin), KRT (keratin), KPRP (keratinocyte proline rich protein), and KRTAP (keratin associated protein), were significantly downregulated by *S. aureus* infection (Figures 4C and 4D). Transcriptome analysis also revealed host cellular responses against bacterial infection including bacterial recognition and inflammatory signaling. A variety of genes involved in dermal- and epidermal-derived inflammation, such as CXCL (C-X-C motif chemokine ligand), CCL (C-C motif chemokine ligand), TSLP (thymic stromal lymphopoietin), IL1 (interleukin 1), and TNF (tumor necrosis factor), were differentially expressed in the damaged skin and tissues with bacterial penetration (Figure 4E).

The expression of inflammatory cytokines and chemokines was significantly upregulated after *S. aureus* infection, indicating that the ALI-skin organoids can capture the cellular responses to infection. Therefore, SA infection of ALI-skin organoids resulted in a compromised skin barrier and an increase in the production of inflammatory cytokines of epidermal and dermal origin.

### Effects of commensal microbiota treatments on *S. aureus*-infected ALI-akin organoids

**Figure 5:**
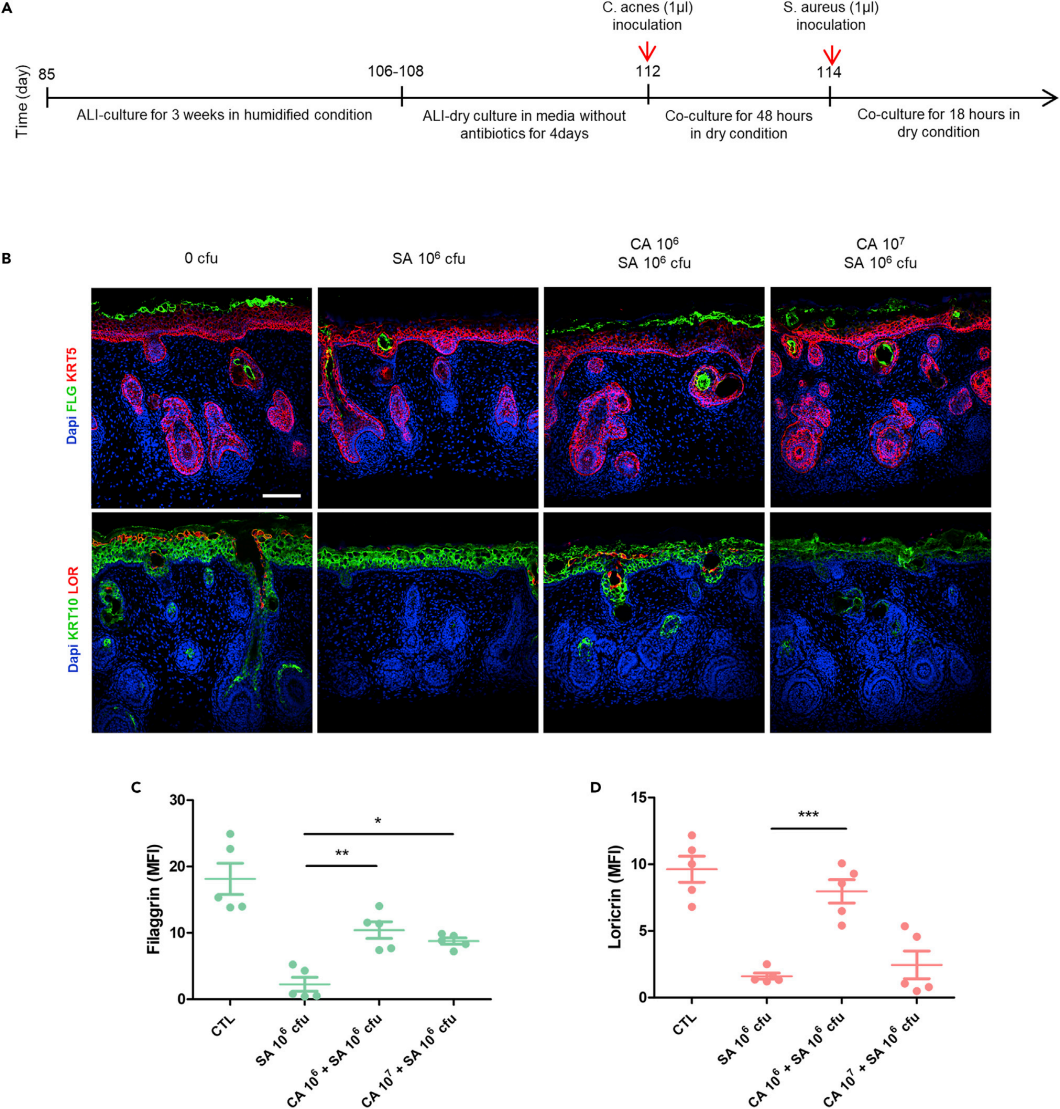
The effect of *Cutibacterium acnes* (*C. acnes*) on ALI-skin organoids infected with *S. aureus* (A) Schematic outline for *C. acnes* pre-treatment on *S. aureus*-infected ALI-skin organoids. (B–D) Epidermal barrier markers were analyzed in 0 CFU (control; PBS only), *S. aureus* (SA) 10^6^ only, *C. acnes* (CA) 10^6^CFU + SA 10^6^ CFU, and CA 10^7^CFU + SA 10^6^ CFU infected ALI-skin organoids. Immunostaining images for KRT5^+^ basal and FLG^+^ cornified epidermal layer marker (top panel) and KRT10^+^ spinous and LOR^+^ granular epithelial layer markers (bottom panel) are shown (B). Scale bars, 50 μm. Quantifications of Filaggrin (C) and Loricrin (D) expression levels are shown. One-way ANOVA with Bonferroni post-hoc test; ∗p < 0.05, ∗∗p < 0.01, ∗∗∗p < 0.001, compared to *S. aureus* (SA) 10^6^; independent replicates = 5. The results are presented as the means ± SEM.

Based on the previous data indicating that *S. aureus* directly causes an AD-like phenotype in the ALI-skin organoids, we aimed to examine whether co-culturing commensal microbiota found on healthy human skin could prevent or reduce the resulting AD phenotype. *C. acnes (C. acnes)* is one of the most common commensal microbiota found on healthy human skin (Byrd et al., 2018; Fitz-Gibbon et al., 2013). Treatment with *C. acnes* was tested for prevention of AD and therapeutic effects on skin barrier function. ALI-skin organoid culture was started around day 85 (Figure 5A) in a 100% humidified incubator for 3 weeks, and then ALI-skin organoids were moved to dry conditions and cultured for an additional 4 days in skin maturation media without antibiotics. The surface of the ALI-skin organoids was pre-treated with different doses (0, 10^6^, and 10^7^ CFU) of *C. acnes* and cultured for 48 h (Figure 5A). Subsequently, 10^6^ CFU of *S. aureus* were inoculated on the *C. acnes* pre-treated ALI-skin organoids (0, SA10^6^ only, CA10^6^ + SA10^6^, and CA10^7^ + SA10^6^ CFU). *C. acnes* and *S. aureus* were co-cultured for 18 h in the dry condition. ALI-skin organoids were immunostained against C. acnes and *S. aureus* to determine the efficacy of the treatment (Figure S5A). To determine whether *C. acnes* had a protective effect on the skin barrier of *S. aureus*-infected ALI-skin organoids, we performed immunostaining for cornified epithelial layer marker, filaggrin, and granular epithelial layer marker, loricrin. As expected, filaggrin and loricrin expressions were diminished in ALI-skin organoids treated only with 10^6^ *S aureus* (Figure 5B). However, ALI-skin organoids pre-treated with 10^6^ CFU of *C. acnes* and infected with *S. aureus* had higher levels of filaggrin and loricrin expression than those treated with *S. aureus* alone (Figure 5B). The ALI-skin organoids were also used to investigate the influence of other commensal microbiota on skin barrier function, including *Staphylococcus epidermidis (S. epidermidis)* (Figure S5B), *Lactobacillus iners* (*L. iners*) (Figure S5C), and mixed microbiota (Figures S5D and S5E), which are found on healthy human skin. Unlike *C. acnes*, these bacteria did not have protective effects on the skin barrier function of *S. aureus-*infected ALI-skin organoids.

## Discussion

In this study, we showed that ihPSC-derived ALI-skin organoids provide a simple yet efficient model system to recapitulate aspects of human skin and model human AD by *S. aureus* skin colonization and infection. We employed several new strategies in this study. First, we improved the current skin organoid culture method by activating the Wnt signaling pathway, resulting in skin organoids of a larger size without off-target differentiation of hyaline cartilages in the tail region. In addition, we modified the skin organoid model by using an ALI culture method. Consequently, enclosed, circular aggregates were transformed into an open skin organoid model whereby skin organoids grow on top of a Transwell culture insert, such that the epidermal layer is exposed to the air. Recently, the ALI culture method has also been adapted into several organoid models. Researchers have generated PSC-derived organoids from various types of organs, including cerebral organoids (Giandomenico et al., 2019) and hepato-biliary-pancreatic organoids (Koike et al., 2019), using ALI and reported improved viability, maturation, and function of organoids. The ALI culture method has also been widely used in a human skin equivalent culture model to induce epidermal stratification and differentiation (Pruniéras et al., 1983; Rosdy and Clauss, 1990). In this study, we also showed that ALI-skin organoids exhibit a dermis and a hypodermis underneath a stratified squamous epidermis layer reminiscent of human skin. Our ALI-skin organoid culture system provides a platform that can be used to model quantitatively various human skin diseases.

Second, the ALI-skin organoids were used to model *S. aureus* skin colonization and infection. *S. aureus*, a common cause of skin infections, is frequently found on the skin of patients with AD, but not on the skin of healthy individuals (Flowers and Grice, 2020). After inoculation of the surface of ALI-skin organoids with *S. aureus*, *S. aureus* was detected in both the epidermis and dermis, indicating that *S. aureus* can actively penetrate the human skin organoid. Immunostaining and transcriptome analysis revealed that *S. aureus* infection caused structural damage to the skin barrier in the epidermal layers of ALI-skin organoids. Infection with *S. aureus* also resulted in epithelial and dermal cell-derived cytokine production. This resembles the AD phenotype described previously (De Benedetto et al., 2011; McAleer and Irvine, 2013).

The skin microbiome plays an important role in regulating skin health and diseases. Recent microbiome studies have demonstrated that commensal bacteria may be an important component of human immunity, protecting tissues from pathogens (Gallo and Nakatsuji, 2011). Third, we demonstrated that *C. acnes*, a commensal component of the skin microbiota, had a preventative and therapeutic effect on the impaired skin barrier of *S. aureus*-infected ALI-skin organoids. We found that pre-treatment with *C. acnes* resulted in increased expression of the filaggrin protein, which has an important function in maintaining the skin epithelial barrier. *C. acnes* has long been thought to be a pathogenic factor for acne; however, it is a major skin commensal that prevents pathogen colonization and invasion. A recent microbiome study also demonstrated that *C. acnes* abundance significantly improved the *S. aureus*-colonized lesioned skin of patients with AD patients, highlighting the crucial role of *C*. *acnes* as a commensal skin microbiota (Francuzik et al., 2018).

### Limitations of the study

The ALI-skin organoid culture system lacks immune cells and vasculature, which create an essential niche during the progression of infectious disease. In this study, it was therefore impossible to replicate the inflammatory component of *S. aureus* infection and the therapeutic effects of commensal microbiota. Future ALI-skin organoid models could include immune cells derived from autologous iPSCs, such as macrophages, T cells, or endothelial cells, to investigate their contributions to skin microbial infections. Another possible limitation is that we used ATCC-purchased microbial strains that may be genetically different from actual human microbiota. In a subsequent study, we intend to investigate the effects of human skin-collected microbial strains on the ALI-skin model system. Collectively, our findings demonstrate that the human ALI-skin organoid platform could be utilized to recapitulate the characteristics of skin diseases, comprehend the underlying pathological mechanisms, and evaluate the efficacy of novel therapies.

## STAR★Methods

### Key resources table

**Table:** 

REAGENT or RESOURCE	SOURCE	IDENTIFIER
**Antibodies**		

Rabbit Anti-KRT5	Abcam	Cat# ab52635; RRID: AB_869890
Mouse Anti-KRT10	Santa Cruz Biotechnology	Cat# sc-23877; RRID: AB_2134668
Mouse Anti-KRT17	Santa Cruz Biotechnology	Cat# sc-393091; RRID: AB_2893343
Mouse Anti-Filaggrin	Santa Cruz Biotechnology	Cat# sc-66192; RRID: AB_1122916
Mouse Anti-Vimentin	Santa Cruz Biotechnology	Cat# sc-6260; RRID: AB_628437
Mouse Anti-COL3A1	Santa Cruz Biotechnology	Cat# sc-27124; RRID: AB_10613985
Rabbit Anti-Loricrin	Abcam	Cat# ab85679; RRID: AB_2134912
Mouse Anti-*Staphylococcus aureus*	Santa Cruz Biotechnology	Cat# sc-58038; RRID: AB_785829
Rabbit Anti-PDGFRα	Cell Signaling Technology	Cat# 3164S
Rabbit Anti-MelanA	Abcam	Cat# ab51061; RRID: AB_880693
Rabbit Anti-TSLP	Abcam	Cat# ab47943; RRID: AB_883272
HCS LipidTOX	Invitrogen	Cat# H34477
Mouse Anti-Claudin4	Invitrogen	Cat# 32-9400; RRID: AB_86919
Mouse Anti-KRT15	Invitrogen	Cat# MA5-11344; RRID: AB_10999819
Mouse Anti-p63	Abcam	Cat# ab735; RRID: AB_305870
Mouse Anti-TFAP	Santa Cruz Biotechnology	Cat# sc-12726; RRID: AB_667767
Mouse Anti-Collagen Type II	MilliporeSigma	Cat# MAB8887; RRID: AB_2260779
Mouse Anti-Fibronectin	Abcam	Cat# ab6328; RRID: AB_305428
Mouse Anti-S100α	Santa Cruz Biotechnology	Cat# sc-53438; RRID: AB_630214
Mouse Anti-E-cadherin	Abcam	Cat# ab1416; RRID: AB_300946
Mouse Anti-Ki67	BD pharmingen	Cat# 550609; RRID: AB_393778
Mouse Anti-hair cortex Cytokeratin/K40	Abcam	Cat# ab16113; RRID: AB_302268
Mouse Anti-NFATc1	Santa Cruz Biotechnology	Cat# sc-7294; RRID: AB_2152503
Mouse Anti-*P. acnes*	MBL life science	Cat# D371-3
Rabbit Anti- alpha smooth muscle Actin	Abcam	Cat# ab5694; RRID: AB_2223021
Rabbit Anti-Sox2	MilliporeSigma	Cat# AB5603; RRID: AB_2286686
Alexa Fluor® 488 goat anti-rabbit IgG (H + L)	Invitrogen	Cat# A11008; RRID: AB_143165
Alexa Fluor® 594 goat anti-rabbit IgG (H + L)	Invitrogen	Cat# A11012; RRID: AB_2534079
Alexa Fluor® 488 goat anti-rabbit IgG (H + L)	Invitrogen	Cat# A11008; RRID: AB_143165
Alexa Fluor® 594 goat anti-rabbit IgG (H + L)	Invitrogen	Cat# A11012; RRID: AB_2534079

**Bacterial and virus strains**		

*Staphylococcus aureus*	American Type Culture Collection	ATCC 12600
*Cutibacterium acnes*	Korean Collection for Type Cultures	KCTC 3314
Human skin tissue	BIOHEAD	Cat# BHF0303210

**Chemicals, peptides, and recombinant proteins**		

Essential 8 medium	Gibco	Cat# A1517001
Essential 6 Medium	Gibco	Cat# A1516401
Vitronectin	Gibco	Cat# A31804
Y-27632	Tocris	Cat# 1254
ReLeSR	Stem Cell Technology	Cat# 100-0484
Accutase	Gibco	Cat# GIB-A11105-01
Growth factor reduced Matrigel	Corning	Cat# 354230
SB431542	Tocris	Cat# 1614/10
Recombinant Human FGF basic	PeproTech	Cat# 100-18B
Recombinant Human BMP-4	Peprotech	Cat# 120-05-100
LDN 193189	Tocris	Cat# 6053
CHIR99021	Tocris	Cat# 4423
Collagen I, rat tail	BD Biosciences	Cat# 354236
Lysogeny Broth (LB) medium	BD Bioscience	Cat# 244620
Reinforced Clostridial Medium	BD Bioscience	Cat# 218081
gelatin/sucrose solution	N/A	https://doi.org/10.1038/s41593-019-0350-2
normal goat serum	Vector Laboratories	Cat# S-1000-20
Bovine serum albumin	GenDEPOT	Cat# A0100-010
Triton X-100	Sigma	Cat# T8787
SYBR™ Green PCR Master Mix	Applied Biosystems	Cat# 4309155
Fluorescent mounting medium	Dako	Cat# S3025

**Critical commercial assays**		

LIVE/DEAD BacLight Bacterial Viability Kit	Thermo Fisher Scientific	Cat# L7012
Click-iT Plus TUNEL Assay Kits for *In Situ* Apoptosis Detection	Thermo Fisher Scientific	Cat# C10617
SuperScript III Reverse Transcriptase	Thermo Fisher Scientific	Cat# 18080085
Rneasy Micro Kit	QIAGEN	Cat# 74004
Easy-spin Total RNA Extraction kit	iNtRON Biotechnology	Cat# 17221
TruSeq Stranded mRNA Sample Prep kit	Illumina	Cat# 20020595
Human TSLP Quantikine ELISA kit	R&D systems	Cat# DTSLP0

**Deposited data**		

RNA-seq data	This paper	GSE211868

**Experimental models: Cell lines**		

Human Induced Pluripotent Stem Cell_CMC003	Korea National Stem Cell Bank	Cat# KSCBi005-A; RRID: CVCL_WR10
Human Induced Pluripotent Stem Cell_CMC0011	Korea National Stem Cell Bank	Cat# KSCBi0018-A; RRID: CVCL_WR33
Skin organoids	Karl R. Koehler laboratory	https://doi.org/10.1038/s41586-020-2352-3

**Oligonucleotides**		

Forward primer for human GAPDH; 5′-GTCAGTGGTGGACCTGACCT-3′	This paper	N/A
Reverse primer for human GAPDH; 5′-TGCTGTAGCCAAATTCGTTG-3′	This paper	N/A
Forward primer for human SOX9; 5′-GAAGCTCGCGGACCAGTACC-3′	This paper	N/A
Reverse primer for human SOX9; 5′-CTGCCCGTTCTTCACCGACT-3′	This paper	N/A
Forward primer for human ACAN; 5′-CTTCTCCGGAATGGAAACGTG-3′	This paper	N/A
Reverse primer for human ACAN; 5′-ACATACCTCCTGGTCTATGTTACAG-3′	This paper	N/A
Forward primer for human COL2A; 5′-CATGAGGGCGCGGTAGAGAC-3′	This paper	N/A
Reverse primer for human COL2A; 5′-TCCCTTTGGTCCTGGTTGCC-3′	This paper	N/A

**Software and algorithms**		

KEGG Mapper	Kyoto Encyclopedia of Genes and Genomes	N/A
GraphPad Prism v.5	Graphpad	https://www.graphpad.com
ImageJ	NIH	https://imagej.nih.gov/ij/download.html

**Other**		

U-bottom low-attachment 96-well plate	Corning	Cat# CLS7007
U-bottom low-attachment 6-well plate	Corning	Cat# CLS3471
12 mm transwell	Corning	Cat# 3460

### Resource availability

#### Lead contact

Further information and requests for resources and reagents should be directed to and will be fulfilled by the lead contact, Kyung-Sun Kang (kangpub@snu.ac.kr).

#### Materials availability

The materials will be available from the lead contact upon request.

### Experimental model and subject details

#### Human induced pluripotent stem cell (hiPSC) culture

Two healthy subject-derived iPSC lines were used in this study. Human iPSCs (CMC lines; CMC003 and CMC011) were obtained from the Korea National Stem Cell Bank (kscr.nih.go.kr). The hiPSC lines had been validated in previous studies (Jeong et al., 2020; Jo et al., 2020). Cultures were tested for mycoplasma and maintained mycoplasma free. hiPSCs were maintained at 37°C with 5% CO_2_ in feeder free conditions. Cells were cultured on Vitronectin-coated (ThermoFisher) 35 mm^2^ dishes in Essential 8 medium (Gibco) supplemented with 10 μM Y-27632c (Tocris). hiPSC cultures were passaged weekly using ReLeSR (Stem Cell Technology). All hiPSC lines were cultured for up to 60–70 passages.

#### Bacterial strains culture conditions

*S. aureus* (ATCC 12600), purchased from the American Type Culture Collection (Manassas, VA, USA), was cultured in Lysogeny Broth (LB) medium (BD Bioscience, San Jose, CA) and grown to the end-exponential growth phase in a shaking incubator at 37°C. *C. acnes* (KCTC 3314), purchased from the Korean Collection for Type Cultures (Jeongeup, Jeollabukdo, Korea), was cultured in Reinforced Clostridial Medium (BD Bioscience, San Jose, CA) supplemented with 0.05% cysteine-HCl at 37°C in an anaerobic jar with a GasPack 100 system (BD Biosciences).

### Methods details

#### Generation of human skin organoids from hiPSCs

Skin organoids were differentiated using a previously published protocol with modifications (Lee et al., 2020). In short, iPSCs were detached with ReLeSR (Stem Cell Technologies) and dissociated into single cells using Accutase (Stem Cell Technologies). Viable cells were counted with a hemocytometer with trypan blue staining. For embryoid body (EB) formation at day 0, approximately 1 × 10^3^ hiPSCs were plated into individual wells of a U-bottom low-attachment 96-well plate (Corning) in Essential 8 (Corning). From day 0 to day 2, 10 μM Y-27632 was added to the media for EB formation. On day 2, the EBs were transferred into individual wells of a new U-bottom low-attachment 96-well plate in E6-based differentiation medium (Thermofisher) containing 2% Matrigel (Corning), 10 μM SB431542 (Tocris), 4 ng/mL FGF (PeproTech), and 15 ng/mL BMP4 (PeproTech). From day 6, 200 ng/mL FGF, 50 μg/mL LDN 193189 (Tocris), and 3 μM Wnt agonist (CHIR99021; Tocris) were added. On days 11–12, skin maturation medium (Lee et al., 2020) was added to each well, and to induce self-assembly of the epidermis, all organoids were transferred into 6-well low-attachment plates (Thermo Fisher Scientific) in 3 mL of skin organoid maturation medium and transferred to a shaker (65rpm; Thermo Fisher). The maturation medium was replaced every 2 to 3 days. No changes were made to the composition of the medium compared with the original protocol (Lee et al., 2020).

#### Air-liquid interface (ALI) skin organoid culture

An acellular collagen layer was prepared at a concentration of 2 mg/mL by neutralizing an acidic collagen solution (BD Collagen I, rat tail, BD Biosciences). After neutralization, 10X PBS was added to make a 1X PBS solution, and distilled water was added to adjust the collagen concentration. A NaOH solution was carefully added to adjust the pH. A total volume of 150 μL of 2% neutralized collagen was deposited on a high-density translucent membrane cell culture insert (0.4 μm pore size) (Corning, NY, USA) in 12-well plates. After 30 min of polymerization in a humidified incubator at 37°C with 5% CO_2_, 600 μL of skin maturation medium was added to the bottom of the insert in a tissue culture 12-well plate. After approximately 85 days of skin organoid culture, each cyst-like skin organoid was cut into four evenly sized portions and placed on the polymerized collagen I-coated Transwell culture inserts. The skin organoid pieces were exposed to the ALI in a humidified incubator at 37°C with 5% CO_2_ for 3–4 weeks. From this point, skin organoid pieces are referred to as ALI-skin organoids. Skin maturation medium was replaced every 2 to 3 days. To induce epidermal maturation, ALI-skin organoids were transferred to an incubator without humidity at 37°C with 5% CO_2_ (dry condition) for 6 more days. The skin medium was replaced daily in dry conditions.

#### Preparation of bacterial strains

Bacterial pellets were collected and re-suspended in sterile PBS solution. The concentrations of bacterial suspensions were calculated with the BD Accuri C6 Plus Flow Cytometer (BD Biosciences) after cell viability staining using the LIVE/DEAD BacLight Bacterial Viability Kit (Thermo Fisher Scientific, Waltham, MA, USA). For co-culture with organoids, *S. aureus* was prepared at 10^5^, 10^6^, and 10^7^ cells/μL concentrations in PBS solution, and *C. acnes* was prepared at 10^6^ and 10^7^ cells/μL concentrations in PBS solution.

#### ALI-skin organoid and bacterial Co-culture

ALI-skin organoids were washed with skin maturation medium without antibiotics once. Prior to inoculation with bacteria, ALI-skin organoids were cultured in skin maturation medium without antibiotics in dry conditions for 6 days. Skin maturation medium was replaced daily. 1 μL of the bacterial suspension was inoculated on top of the ALI-skin organoid and allowed to dry. The control was treated with the PBS vehicle only. The ALI-skin organoid and bacteria were co-cultured in an incubator without humidity at 37°C with 5% CO_2_ (dry condition) for 18, 24, or 48 h, depending on the experiment.

#### Human skin sample preparation

Human thigh skin samples were obtained from healthy female (63 years of age) through BIOHEAD (Seoul, Republic of Korea, BHF0303210). The skin samples were fixed with 4% paraformaldehyde (PFA) in PBS and embedded in a gelatin/sucrose solution for immunohistochemistry.

#### Cryopreservation and immunohistochemistry

For immunostaining, skin organoids were fixed with 4% paraformaldehyde (PFA) in PBS overnight at 4°C and cryoprotected by incubating in 30% sucrose overnight followed by embedding in a gelatin/sucrose solution^19^. Tissue blocks were frozen with liquid nitrogen and then stored at −80°C in a sealed container. Frozen blocks were sliced to a thickness of 12 μm. Antigen retrieval was carried out at 95°C for 3 min using 10 mM sodium citrate. Sections were blocked for 1 h at room temperature in blocking buffer, which consisted of 5% normal goat serum, 1% BSA, and 0.25% Triton X-100 in PBS. Primary antibodies were diluted in blocking buffer overnight at 4°C. PBS was used to wash-off the primary antibodies and the sections were incubated with secondary antibodies in 1% bovine serum albumin and 0.25% Triton X-100 in PBS for 1 h. After incubation with secondary antibodies, sections were washed three times with PBS for 15 min. The following primary antibodies were used for immunohistochemistry:

#### Quantitative reverse transcription PCR analysis

Quantitative reverse transcription PCR (qPCR) was performed with RNA lysates harvested from cultured cells. Total RNA was isolated with a RNeasy kit (Qiagen), and cDNA was prepared using reverse transcriptase III (Thermo Fisher Scientific), according to the manufacturer’s instructions. Real-time PCR was performed using the SRBR Green Master Mix (Thermo Fisher Scientific), and detection was achieved using the Step One Plus Real-time PCR system thermocycler (Applied Biosystems). Expression of target genes was normalized to that of glyceraldehyde-3- phosphate dehydrogenase (GAPDH).

#### RNA extraction and sequencing analysis

The total RNA was extracted from three biological replicates of ALI-skin organoids after treatment with *S. aureus* (10^6^ CFU) for 18 h using the Easy-spin Total RNA Extraction kit (iNtRON Biotechnology, Seoul, Korea). The quality confirmation, reverse transcription, and sequencing processes were conducted by Macrogen Inc. (Seoul, Korea) based on the Illumina sequencing platform and protocols. The cDNA library was performed using the TruSeq Stranded mRNA Sample Prep kit (Illumina, Inc., San Diego, CA). The libraries were sequenced on a HiSeq2500 platform to generate paired-end 100 bp sequence reads. The reads were mapped and quantified using the Bowtie2 (version 2.3.4.1) aligner and HISAT2 (version 2.1.0) with the human genome (hg19/GRCh37). Differentially expressed genes (DE-Gs) were called using the DESeq2 packages. Of the 46,427 genes identified, 26,458 with at least one zero fragments per kilobase of transcript per million mapped reads were excluded, which left 19,969 genes to be processed for DE-Gs analysis. DE-Gs were defined as showing >2-fold changes in expression, with the adjusted p value < 0.05. The functional annotation and Gene Ontology Enrichment analysis was performed using g:Profiler. The gene-set enrichment test and the mapping of KEGG pathways were performed using KEGG Mapper.

#### TSLP ELISA

The level of TSLP in the supernatant was measured by the Human TSLP Quantikine ELISA kit (R&D Systems; Minneapolis, MN, DTSLP0) according to the manufacturer’s instructions.

### Quantification and statistical analysis

Data are presented as mean ± SEM, unless otherwise indicated. Analyses were performed using unpaired two-tailed Student’s t-tests, one-way ANOVA or two-way ANOVA followed by the Bonferroni’s test were performed to compare data for multiple groups throughout the experiments. All statistical analyses were performed using GraphPad Prism v.5 software (Graphpad Software Inc.), ∗∗∗p < 0.001, ∗∗p < 0.01, and ∗p < 0.05 were considered statistically significant herein; and ns indicates not significant.

## Data Availability

• RNA-seq data have been deposited at Gene Expression Omnibus (GEO: GSE211868) and are publicly available as of the date of publication. • This paper does not report original code. • Any additional information required to reanalyze the data reported in this paper is available from the lead contact upon request.
