# Supplementary material for: Wnt-activating human skin organoid model of atopic dermatitis induced by Staphylococcus aureus and its protective effects by Cutibacterium acnes
Source: iScience. 2022 Sep 16;25(10):105150. doi: 10.1016/j.isci.2022.105150 (PMC9526179; doi:10.1016/j.isci.2022.105150)
Supplement: Document S1. Figures S1–S5 [file mmc1.pdf]

iScience, Volume *25*

Supplemental information

Wnt-activating human skin organoid model of atopic dermatitis induced by *Staphylococcus aureus* and its protective effects by *Cutibacterium acnes*

Song-yi Jung, Hyun Ju You, Min-Ji Kim, GwangPyo Ko, Seunghee Lee, and Kyung-Sun Kang

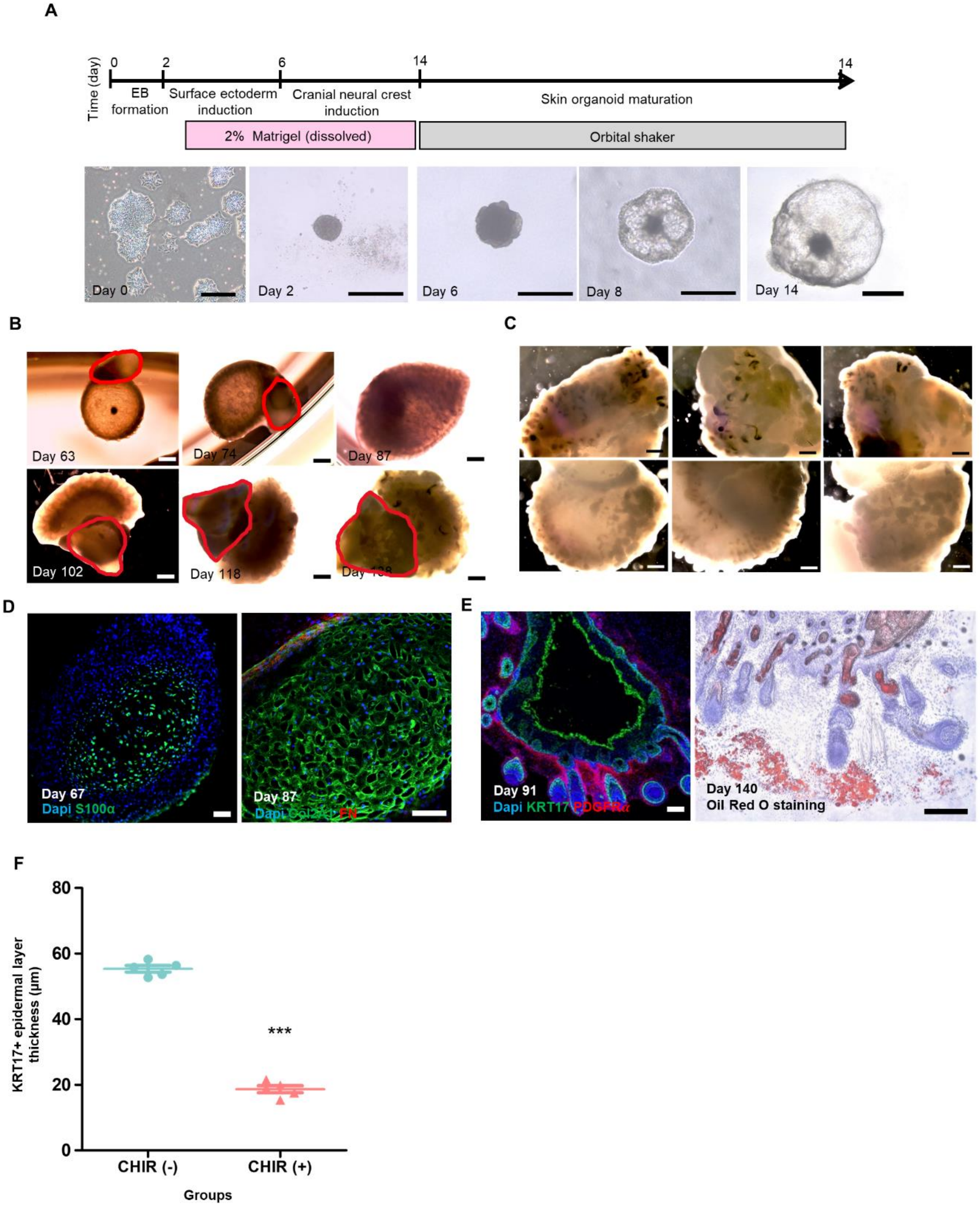

Figure S1. Description of the skin organoid protocol and generation of human iPSC-derived skin organoids. Related to Figure 1.

(A) Schematic overview and timeline of the skin organoid differentiation protocol based on the Lee *et al.* culture method and phase contrast images of skin organoids in early stages of development (day 1 to 14). Scale bar 500μm.

(B) Bright-field images of skin organoids in later stages of development (days 63 to 138). The red circles represent hyaline cartilage. Scale bar 500μm. Skin organoids were cultured based on the protocol of Lee *et al.*'s culture method.

(C) Representative bright-field images of skin organoids containing pigmented (top panel) or albino (bottom panel) hair follicles at day 140. Scale bar, 500μm.

(D) Immunostaining for S100α in green on the left panel and Col2A1 in green and FN (fibronectin) in red on the right panel at day 67. Scale bars, 50μm.

(E) Immunostaining for $KRT17^+$ epithelial layer and $PDGFR\alpha^+$ dermal layer (green and red, respectively) in day 91 skin organoid on the left. Scale bars, 50μm and on the right, oil red O staining at day 140 of differentiation of skin organoid. Scale bars, 200μm.

(F) Quantification of $KRT17^+$ epidermal layer thickness in skin organoids with or without CHIR treatment at day 88. The epidermal layer thickness of the organoid was measured using Image J. (Fig. 1D; CHIR (+) versus CHIR (-)). One-way ANOVA with Bonferroni post-hoc test; ***$p < 0.001$, independent replicates = 5

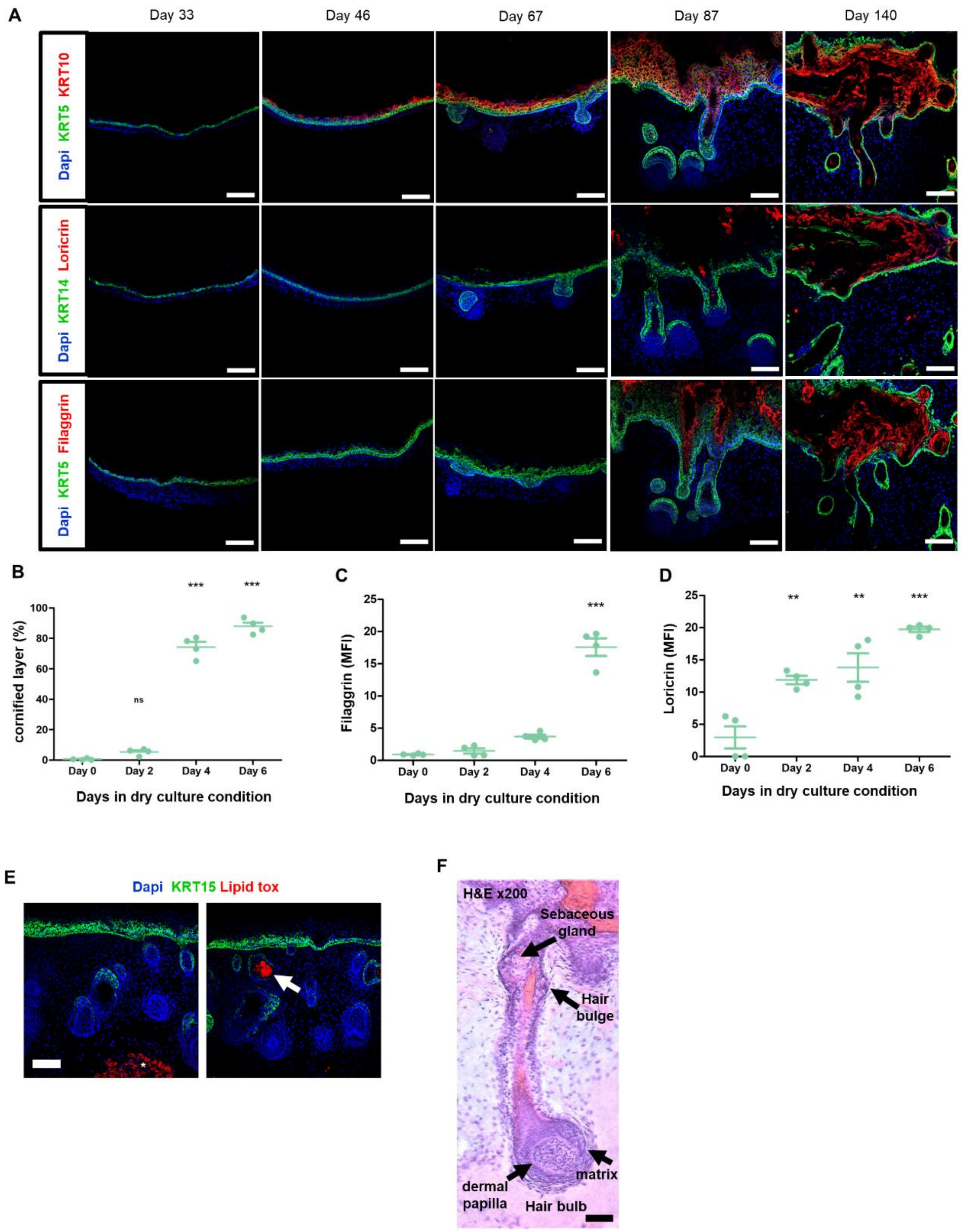

Figure S2. Generation of ALI-skin organoids. Related to Figure 2.

(A) Time course of epidermal layer development in whole skin organoid. Skin organoids were cultured based on the protocol of Lee *et al*.'s culture method. Immunohistochemical analysis for KRT5 and KRT10 (green and red, respectively), KRT14 and Loricrin (green and red, respectively), and KRT5 and filaggrin (green and red, respectively) at different time points of culture (day 33, 46, 67, 87, and 140). Scale bars, 50 μm.

(B) The quantification of cornified stratum formation in 0, 2, 4, and 6 days in dry culture conditions as the length of the corneum formed on the epidermal layer using Image J is shown. (Fig.1B; one-way ANOVA with Bonferroni post-hoc test; Compared to Day 0; independent replicates = 4).

(C-D) Filaggrin (C) and Loricrin (D) expression levels (MFI) were quantified in dry culture conditions at 0, 2, 4, and 6 days (Fig. 1C; one-way ANOVA with Bonferroni post-hoc test; compared to Day 0; independent replicates = 4).

(E) The hypodermal layer (asterisk; left) and sebaceous gland lipid (arrow; right) in ALI-skin organoids are characterized by lipid tox staining in red and basal epithelial layer marker (KRT5) in green. Scale bars, 50 μm.

(F) H&E staining of a hair follicle structure in an ALI-skin organoid is shown. Scale bar, 10μm

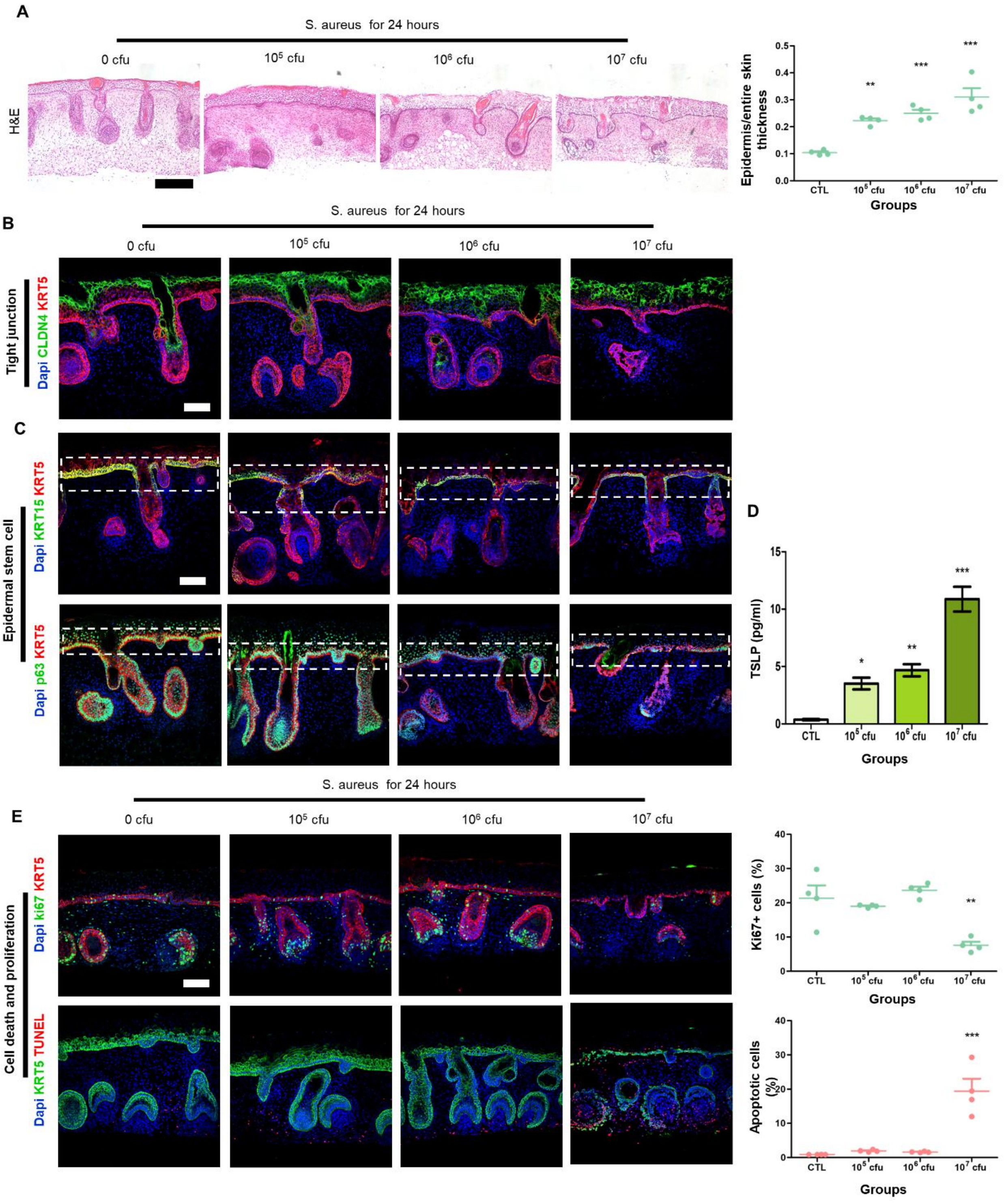

Figure S3. Modeling AD by *S. aureus* colonization and infection of ALI-skin organoids. Related to Figure 3.

(A) H&E staining of 0 CFU, 10^5 CFU, 10^6 CFU, or 10^7 CFU *S. aureus* infected ALI-skin organoids is shown on the left. Scale bars, 100 μm. The quantification of structural damage in the epidermal layers in each group is shown on the right. The thickness of the damaged epidermis was divided by the thickness of the entire skin, measured and quantified by Image J. One-way ANOVA with Bonferroni post-hoc test; *$p < 0.05$, ***$p < 0.001$, compared to control; independent replicates = 4

(B) Immunostaining for Claudin 4 and KRT5 (green and red, respectively) in ALI-skin organoids infected with 0 CFU, 10^5 CFU, 10^6 CFU, or 10^7 CFU *S. aureus*.

(C) Immunostaining for $KRT15^+KRT5^+$ (top panel) and $p63^+KRT5^+$ (bottom panel) epidermal stem cells in ALI-skin organoids infected with 0 CFU, 10^5 CFU, 10^6 CFU, or 10^7 CFU *S. aureus*. The epidermal stem cell regions are indicated in dashed boxes. Scale bars, 50 μm.

(D) After 24 hours, TSLP is measured in culture supernatants of *S. aureus*-infected ALI-skin organoids using an ELISA. Values represent the mean ±SEM. Statistical analysis was calculated using one-way ANOVA with Bonferroni Bonferroni post-hoc test; *$p < 0.05$, **$p < 0.01$, ***$p < 0.001$, compared to control (0 CFU); independent replicates = 3.

(E) Analysis of proliferation and apoptotic cell death in ALI-skin organoids infected with 0 CFU, 10^5 CFU, 10^6 CFU, or 10^7 CFU *S. aureus*. Immunostaining images for $Ki67^+$ proliferating cells in green (left) and quantification of the percentage of $ki67^+$cells in total dapi stained cells per frame (right) are shown on the top panel. Immunostaining images for TUNEL stained apoptotic cells in red (left) and quantification of the percentage of TUNEL stained cells in the total of dapi stained cells per frame (right) are shown on the bottom panel. One-way ANOVA with Bonferroni post-hoc test; **$p < 0.01$, ***$p < 0.001$, compared to control (0 CFU); ($n = 4$)

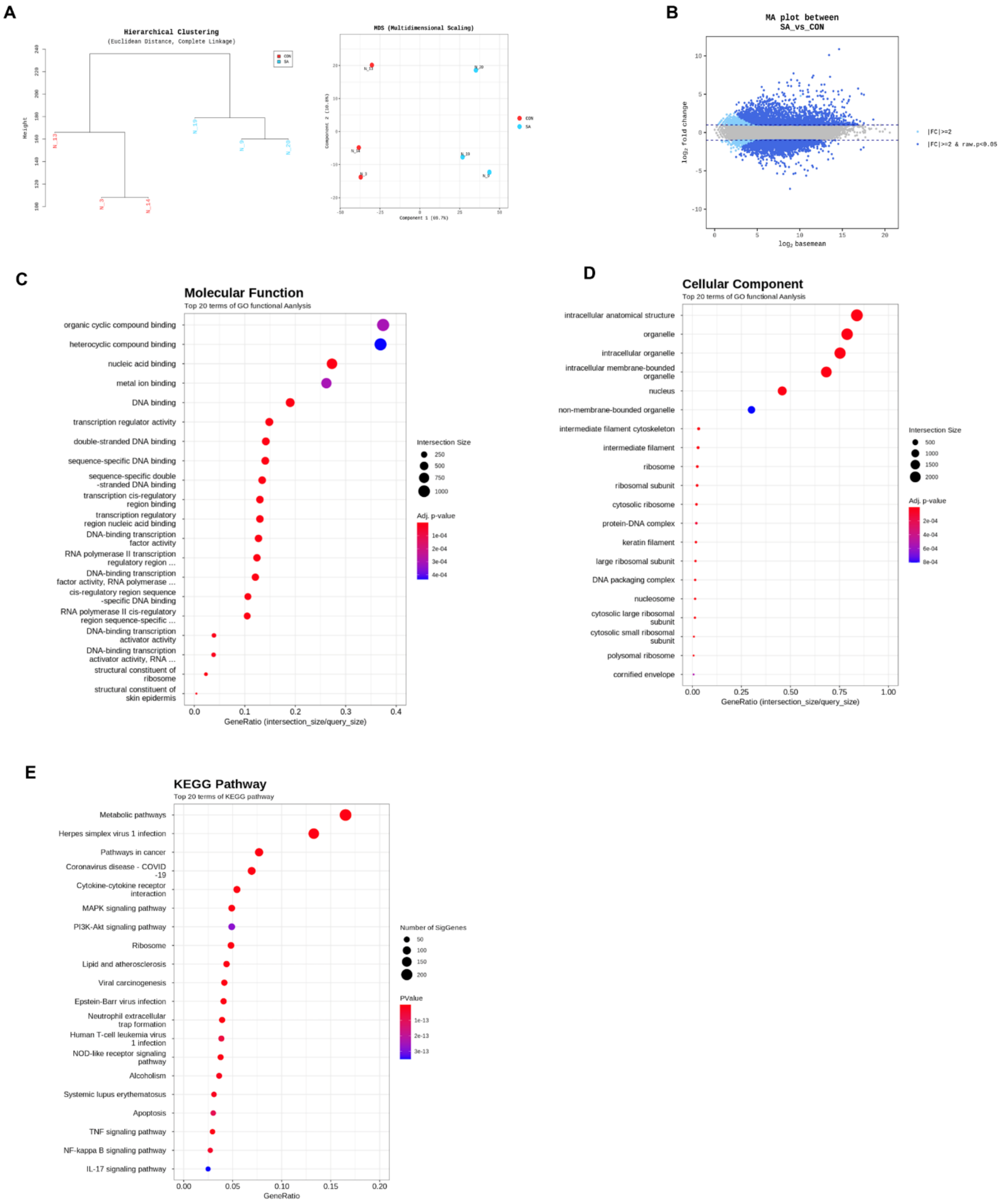

Figure S4. Transcriptome analysis of *S. aureus*-infected ALI-skin organoids. Related to Figure 4.

(A) Description of hierarchical clustering and MDS analysis of distinct gene expression in the *S. aureus*-treated ALI-skin organoids in blue and untreated controls in red.

(B) A volcano plot is used to visualize the differentially expressed genes (DEGs). Of the 4,255 DEGs, 2,162 genes were upregulated and 2,093 genes were downregulated by *S. aureus* infection (the blue dots indicate DEGs with an adjusted p value < 0.05).

(C-E) The DEGs were classified based on the GO terms of biological process, molecular function, and cellular component, and KEGG pathway analysis of differentially expressed genes.

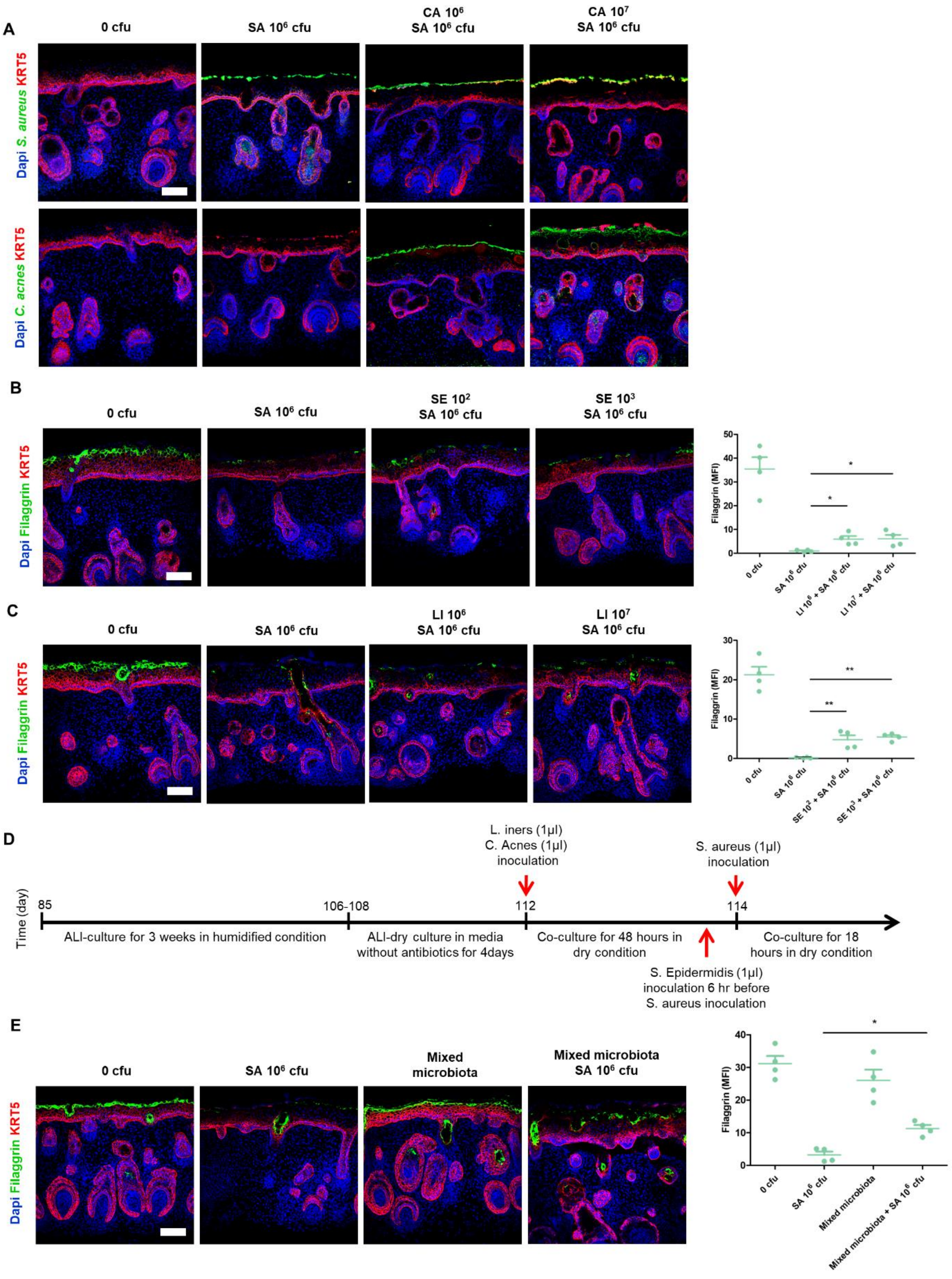
A
0 cfu
SA 10^6 cfu
CA 10^6
SA 10^6 cfu
CA 10^7
SA 10^6 cfu
Dapi S. aureus KRT5
Dapi C. acnes KRT5
B
0 cfu
SA 10^6 cfu
SE 10^2
SA 10^6 cfu
SE 10^3
SA 10^6 cfu
Dapi Filaggrin KRT5
Filaggrin (MFI)
0 cfu
SA 10^6 cfu
LI 10^6 + SA 10^6 cfu
LI 10^7 + SA 10^6 cfu
C
0 cfu
SA 10^6 cfu
LI 10^6
SA 10^6 cfu
LI 10^7
SA 10^6 cfu
Dapi Filaggrin KRT5
Filaggrin (MFI)
0 cfu
SA 10^6 cfu
SE 10^2 + SA 10^6 cfu
SE 10^3 + SA 10^6 cfu
D
L. iners (1µl)
C. Acnes (1µl)
inoculation
S. aureus (1µl)
inoculation
Time (day)
85
106-108
112
114
ALI-culture for 3 weeks in humidified condition
ALI-dry culture in media
without antibiotics for 4days
Co-culture for 48 hours in
dry condition
Co-culture for 18
hours in dry condition
S. Epidermidis (1µl)
inoculation 6 hr before
S. aureus inoculation
E
0 cfu
SA 10^6 cfu
Mixed
microbiota
Mixed microbiota
SA 10^6 cfu
Dapi Filaggrin KRT5
Filaggrin (MFI)
0 cfu
SA 10^6 cfu
Mixed microbiota
Mixed microbiota + SA 10^6 cfu

Figure S5. Effects of commensal microbiota treatments on *S. aureus*-infected ALI-skin organoids. Related to Figure 5.

(A) Immunostaining for *S. aureus* (top panel; green) and *C. acnes* (bottom panel; green) to visualize bacteria infection and $KRT5^{+}$ basal epithelial layer maker (red) in ALI-skin organoids infected with 0 CFU (control; PBS only), *S. aureus* (SA) 10^6 only, *C. acnes* (CA) 10^6 CFU + SA 10^6 CFU, and CA 10^7 CFU + SA 10^6. Scale bars, 50 μm.

(B) Immunostaining images for $filaggrin^{+}$ cornified epidermal layer in green and $KRT5^{+}$ basal epidermal layer in red in ALI-skin organoids infected with 0 CFU, SA 10^6 only, *S. epidermidis* (SE) 10^2 CFU+ SA 10^6 CFU, and SE 10^3 CFU+ SA 10^6 CFU (left). Scale bars, 50 μm. The quantification of filaggrin expression levels determined by mean fluorescence intensity (MFI) is shown on the right. One-way ANOVA with Bonferroni post-hoc test; $^*p < 0.05$, $^{**}p < 0.01$, $^{***}p < 0.001$, compared to SA 10^6 CFU; independent replicates = 4

(C) Immunostaining images for $filaggrin^{+}$ cornified epidermal layer in green and $KRT5^{+}$ basal epidermal layer in red in ALI-skin organoids infected with 0 CFU, SA 10^6 only, *Lactobacillus iners* (LI) 10^6 CFU+SA 10^6 CFU, and LI 10^7 CFU+SA 10^6 CFU are shown (left), and the quantification of filaggrin expression levels (MFI) is shown (right).

(D-E) Schematic outline for mixed microbiota (CA 10^6, LI 10^6, and SE10^2 CFU) pre-treatment on *S. aureus* (10^6 CFU) infected ALI-skin organoids (D), and immunostaining images for filaggrin (green) and KRT5 (red) in 0 CFU, SA 10^6 only, mixed microbiota, and mixed microbiota + SA 10^6 CFU infected ALI-skin organoids (left) and the quantification of filaggrin expression levels (MFI; right) are shown (E).
